# Substrate stiffness modulates extracellular vesicles’ release in a triple-negative breast cancer model

**DOI:** 10.20517/evcna.2024.47

**Published:** 2024-09-29

**Authors:** Beatrice Senigagliesi, Otmar Geiss, Stefano Valente, Hendrik Vondracek, Nicola Cefarin, Giacomo Ceccone, Luigi Calzolai, Laura Ballerini, Pietro Parisse, Loredana Casalis

**Affiliations:** ^1^Neuroscience Area, Scuola Internazionale Superiore di Studi Avanzati, Trieste 34136, Italy.; ^2^Nano-Innovation Laboratory, Elettra-Sincrotrone Trieste S.C.p.A., Trieste 34149, Italy.; ^3^Institute for Health and Consumer Protection, European Commission - Joint Research Centre, Ispra 21027, Italy.; ^4^Department of Physics, University of Trieste, Trieste 34127, Italy.; ^5^Istituto Officina dei Materiali, Consiglio Nazionale delle Ricerche, CNR-IOM, Trieste 34149, Italy.

**Keywords:** Cancer-derived extracellular vesicles, mechano-transduction, atomic force microscopy, nanoparticle tracking analysis, asymmetric flow field flow fractionation-multi-angle light scattering

## Abstract

**Aim:**

The microenvironment effect on the tumoral-derived Extracellular Vesicle release, which is of significant interest for biomedical applications, still represents a rather unexplored field. The aim of the present work is to investigate the interrelation between extracellular matrix (ECM) stiffness and the release of small EVs from cancer cells. Here, we focus on the interrelation between the ECM and small extracellular vesicles (sEVs), specifically investigating the unexplored aspect of the influence of ECM stiffness on the release of sEVs.

**Methods:**

We used a well-studied metastatic Triple-Negative Breast Cancer (TNBC) cell line, MDA-MB-231, as a model to study the release of sEVs by cells cultured on substrates of different stiffness. We have grown MDA-MB-231 cells on two collagen-coated polydimethylsiloxane (PDMS) substrates at different stiffness (0.2 and 3.6 MPa), comparing them with a hard glass substrate as control, and then we isolated the respective sEVs by differential ultracentrifugation. After checking the cell growth conditions [vitality, morphology by immunofluorescence microscopy, stiffness by atomic force microscopy (AFM)], we took advantage of a multi-parametric approach based on complementary techniques (AFM, Nanoparticle Tracking Analysis, and asymmetric flow field flow fractionation with a multi-angle light scattering detector) to characterize the TNBC-derived sEV obtained in the different substrate conditions.

**Results:**

We observe that soft substrates induce TNBC cell softening and rounding. This effect promotes the release of a high number of larger sEVs.

**Conclusion:**

Here, we show the role of ECM physical properties in the regulation of sEV release in a TNBC model. While the molecular mechanisms regulating this effect need further investigation, our report represents a step toward an improved understanding of ECM-cell-sEVs crosstalk.

## INTRODUCTION

In the past decade, nano-sized (from 30 up to 5,000 nm in diameter) extracellular vesicles (EVs) that serve as an inter-cellular signaling system released by various cell phenotypes have attracted growing interest from the scientific community. These nano-carriers delimited by a lipid bilayer can contribute, together with non-vesicular particles, to the transfer of functional cargos (e.g., proteins, nucleic acids, and lipids) from donor cells to target cells^[1]^. In particular, the small EVs (sEVs, 30 to 200 nm)^[2]^ have recently received increasing attention. The sEV content, which reflects the molecular fingerprint of the parental cell, has been shown to have regulatory effects in target cells both in physiological and pathological conditions, including cancer^[3-5]^. Considering their intrinsic properties, engineered EVs can be exploited for therapeutic purposes^[6,7]^ and as potential diagnostic, prognostic, and predictive biomarkers in cancer and other diseases^[8,9]^.

Triple-negative breast cancer (TNBC) is the most aggressive breast cancer subtype with a poor prognosis, characterized by high aggressiveness and absence of targetable receptors^[10]^. Nowadays, chemotherapy is the main treatment in both early and advanced stages of TNBC^[11]^, and unfortunately, approximately 80% of TNBC patients show an incomplete response to the therapy, disease recurrence, and metastasis formation after surgery^[12,13]^. Thus, additional studies aimed at further understanding the mechanisms of invasion, metastasis, and resistance to therapy are needed to set up effective therapies for TNBC. It has been reported that TNBC-derived sEVs can mediate cell-cell and cell-matrix communication by transferring oncogenic molecules that promote proliferation, migration, invasion, and metastatic spreading in target cells^[14-18]^.

Many studies demonstrated that tumors consist not only of cancer cells but also of a significantly altered surrounding, identified as tumor microenvironment, which plays crucial roles in tumoral development, progression, metastasis formation, and response to therapy^[19-21]^. The tumor microenvironment (TME) comprises multiple cell types (e.g., fibroblasts, endothelial cells, adipocytes, and immune cells)^[20,22]^ and a non-cellular component composed of polymeric proteins and accessory molecules, named extracellular matrix (ECM)^[22,23]^. The ECM is a scaffold of fibrillar proteins, accessory proteins and molecules (e.g., collagen, laminin, fibronectin, proteoglycans) that provides structural and biochemical support for cells^[22]^. The crosstalk between the epithelial and surrounding environment ensures the normal development and differentiation of the mammary gland^[24]^. Many studies underlined how tumor cells adapt to the ECM properties; in particular, the biochemical and biophysical properties of the ECM are able to influence cell plasticity (and vice versa)^[25]^, migration and invasion of cancer cells^[26-29]^. However, the way the stiffness of the microenvironment could affect the tumoral-derived EV release has been investigated in the last years with scattered results and still represents a rather unexplored field^[30-32]^. Here, we use TNBC cells as a model to study the sEV release from MDA-MB-231 cells plated on substrates of different stiffness. The sEVs released under the different conditions have been analyzed through a multi-parametric approach based on orthogonal techniques [high-resolution imaging through Atomic Force Microscopy (AFM)], light scattering technique through nanoparticle tracking analysis (NTA), and microfluidic method through asymmetric flow field flow fractionation with a multi-angle light scattering detector (AF4-MALS) to provide a complete assessment of their physical-chemical properties.

## METHODS

### Fabrication of polydimethylsiloxane substrates

Polydimethylsiloxane (PDMS) substrates at different elastic modulus were fabricated. Silicone elastomer base and curing agent (Sylgard® 184, Dow corning) were mixed at ratios of 10:1 and 50:1, respectively; the mixtures were degassed, poured on the petri dish, and then heated for 3 h at 80 °C. The cured mixtures were exposed to UV for 30 min. To improve the biocompatibility and stabilize cell adhesion, petri dishes with or without PDMS were incubated with collagen [0.1 mg/mL of Collagen I type from rat tail, Gibco, A1048301, in phosphate-buffered saline (PBS)] for 4 h and then washed twice with PBS. Dish without PDMS was used as negative control (referred to as CTRL).

### Cell cultures and small extracellular vesicle isolation

The TNBC MDA-MB-231 cells, kindly provided by the laboratory of Prof. G. Del Sal (Dept. Life Sciences, University of Trieste, Italy), were cultivated in DMEM (Dulbecco’s Modified Eagle’s Medium High Glucose with Sodium Pyruvate with L-Glutamine, EuroClone, ECM0728L) supplemented with 10% Fetal Bovine Serum (FBS) (Fetal Bovine Serum South America origin EU, EuroClone, ECS0180L) and 1% Penicillin/Streptomycin (100X, EuroClone, ECB3001D) in humidified 5% CO_2_ incubator at 37 °C; depending on their confluence, the cells were split every 2-3 days. The phenotype of the cancer cells was regularly monitored; regular checks for Mycoplasma contamination were performed. Culture conditions, as indicators of cell function, were regularly monitored by checking viability, proliferation status, and confluence (maintaining similar confluence). For sEV isolation, cells (3 × 10^6^) were grown in 150 mm collagen-coated petri dishes in DMEM with 10% of ultrafiltrated EV-depleted FBS (UF-dFBS). FBS was centrifuged with Amicon ultra-15 centrifugal filters (Ultracel-PL PLHK, 100 kDa cutoff, Merck Millipore, #UFC9100) for 40 min at 4,000 g in order to obtain UF-dFBS by removing serum EVs and large contaminating proteins, following the protocol provided by Kornilov *et al*.^[33]^. The cellular vitality of MDA-MB-231 cells grown with UF-dFBS for 1, 2 or 3 days was tested [histogram shown in Supplementary Figure 1] through MTT assay and revealed that cells can be grown in this condition for up to 2 days. The medium containing the vesicles released from the cells during the 2 days was collected. The medium was then centrifuged at 300 g for 10 min at 4 °C to remove cells and cellular debris remaining in the pellet. A 0.2 µm filter was then used to filter the supernatant, which was then transferred to Amicon ultra-15 centrifugal filters (Ultracel-PL PLHK, 100 kDa cutoff, Merck Millipore, UFC9100) to centrifuge at 4,000 g for 40 min at 4 °C. The concentrated samples were transferred to ultracentrifuge tubes (Beckman Coulter, 361623) along with PBS to reach the final volume and were ultracentrifuged at 120,000 g for 120 min at 4 °C (rotor 70.1 Ti, k factor 36, Beckman Coulter, Brea, CA, USA). Finally, after removing the supernatant, the pellet was resuspended in PBS and the sEVs were stored at + 4 °C or -80 °C for short periods.

### AFM force spectroscopy

A Smena AFM (NT-MDT Co., Moscow, Russia) on an inverted fluorescence microscope (Nikon Eclipse Ti-U) was used for force spectroscopy analysis of both substrates and cells. A 20 µm diameter-spherical silicon tip glued on a HA-NC Etalon cantilever (k = 5 N/m) or a Novascan calibrated cantilever (k = 0.063 N/m) was used to collect the overall stiffness of the substrates and cell, respectively. Incubation in 4% paraformaldehyde for 20 min was used to fix the cells, then washed in PBS and stained with DAPI. Cells were measured in PBS buffer with 1% penicillin/streptomycin at room temperature (RT). Although fixation in PFA can induce altered cell rigidity^[34]^, it is widely reported that relative changes in stiffness after treatments remain statistically significant despite being fixed^[35]^. In addition, fixation prevents damage due to cell aging and is necessary for immunofluorescence investigations. Force spectroscopy analyses were carried out at constant velocity (2 μm/s), with a maximal indentation of 0.5 μm, and applying to the sample a force of 1-2 nN. Elastic modulus values (E, kPa) were calculated by fitting the obtained force-displacement curves with the Hertz model (using AtomicJ® software)^[36]^.

### Immunofluorescence

Immunofluorescence images were acquired by using an Inverted Research Microscope Eclipse Ti2, Nikon microscope equipped with an epifluorescence illuminator and a highly sensitive scientific Complementary Metal-Oxide-Semiconductor (sCMOS) camera (Prime BSI, Teledyne Photometrics). Cells were fixed (as explained above), permeabilized with 0.5% PBS-Tween® 20 (Sigma-Aldrich) for 10 min and 0.1% PBS-Tween® 20 for 5 min (three times). Subsequently, unspecific sites of cells were blocked in 1% BSA in 0.1% PBS-Tween® 20 for 60 min. Cells were incubated with Alexa Fluor 594 phalloidin (Invitrogen, A12381) in a humidified chamber or under agitation at room temperature for 45 min to visualize and quantify F-actin. Nuclei were stained with DAPI (Sigma Aldrich). Images were analyzed using ImageJ®.

### Cell vitality assay

Cell vitality of MDA-MB-231 cells grown on collagen-coated PDMS substrates was tested using the Live and Dead Cell Assay (Abcam, ab115347). This assay stain solution is a mixture of two fluorescent dyes that differently label live and dead cells. Live cells were identified based on the intracellular esterase activity (green), while dead cells with compromised plasma membranes were marked by red dye staining. The Live and Dead dyes diluted in PBS were directly added to the medium cell culture media for an incubation time of 15 min. Labeled cells were analyzed via epifluorescent microscopy (same setup described above).

### AFM

A commercially available microscope (MFP-3D Stand Alone AFM from Asylum Research, Santa Barbara, CA) and a BL-AC40TS-C2 cantilever (Olympus Micro Cantilevers, nominal elastic constant 0.09 N·m^-1^ and resonant frequency 110 kHz) were used to acquire the AFM images. Measurements were carried out in liquid (PBS) at room temperature and in dynamic AC mode. For AFM imaging of sEVs, a drop of Poly-L-Lysine (Sigma-Aldrich) was incubated for 15 min at room temperature on a freshly cleaved muscovite mica sheet (Ruby Muscovite Mica Scratch Free Grade V-1, Nanoandmore GMBH, USA). The Poly-L-Lysine excess was then rinsed twice with Milli-Q H_2_O. A drop of the vesicle sample suspension was added to the poly-lysine-coated mica surface for 15 min at room temperature in order to enable the sEVs to bind to the surface via electrostatic binding interactions. For each sample, at least five images were acquired with a scan size of 10 µm × 10 µm and a resolution of 1,024 pixels × 1,024 pixels (pixel size ~ 10 nm × 10 nm) (triplicate experiment). The AFM images were analyzed using the grain analysis of the Gwyddion® software. To take into account the convolution effect of the tip (roughly 10 nm radius), we measured the size distribution after setting a threshold of 10 nm above the mica surface; therefore, we analyzed the vesicle heights and diameters above this threshold. In this way, the possible tails of the convolution with the tip shape are not considered, giving a fair estimation of the real lateral size of the EVs. Three independent experiments were performed.

### NTA

A Nanosight LM10 setup (NanoSight Ltd., U.K.) equipped with a 20 mW red laser operating at 655 nm was used to obtain the concentration and particle size distribution of sEVs derived from MDA-MB-231 cells. Each sample, once properly diluted in PBS, was recorded for 60 s using a Marlin F-033B ASG CCD Camera (Allied Vision Technologies GmbH, Germany), and a PL L 20/0.40 objective, adjusting shutter and gain of the camera to track the nanoparticles correctly. Temperature was monitored during the entire measuring period. Vesicle size distribution and their estimated concentration were obtained from the given raw data files with the proprietary software (NTA 2.0) without further elaboration. Three independent experiments were performed.

### Asymmetric flow field flow fractionation with a multi-angle light scattering detector

The asymmetric flow field flow fractionation (AF4) system used for the analysis of sEVs was composed of an Eclipse Dualtec separation system (Wyatt Technology Europe GmbH, Dernbach, Germany) and an Agilent 1260 Infinity high-performance liquid chromatograph (Agilent Technologies, Santa Clara, USA) equipped with a degasser (G1322A), an isocratic pump (G1310B), an autosampler (G1329B) and a multi-wavelength detector (G1365C). The AF4 system was coupled to a DAWN 8+ HELEOS II Multi‐angle light scattering (MALS) detector operating with a 658 nm laser (Wyatt Technology Europe). In the Eclipse SC separation channel, regenerated cellulose membranes (10 kDa) and a spacer height of 350 μm were used. The AF4 separation flow parameters are included in Table 1. Samples were diluted in filtered (0.22 µm) PBS and the same buffer solution was used as control in the reference cell. Three independent experiments were performed.

**Table 1:** AF4 separation setting

**Step**	**Time** **(min)**	**Detector flow** **(mL·min^-1^)**	**Cross flow** **(mL·min^-1^)**	**Focus flow** **(mL·min^-1^)**
Elution	0-3	0.5	0	0
Focus	3-5	0.5		1
Focus + inject	5-10	0.5		1
Elution	10-50	0.5	1-0 (linear gradient)	0
Elution	50-60	0.5	0	0

AF4: Asymmetric flow field-flow fractionation.

### Scanning electron microscopy

A Zeiss Supra40 acquire scanning electron microscopy (SEM) was used to acquire SEM images of EVs. Images were taken at low accelerating voltage (5 keV) by capturing the secondary electrons. Acetone and isopropanol were used to clean the silica slide and a drop of Poly-L-lysine (Sigma-Aldrich) was then added to the top to facilitate the capture of the vesicles based on the electrostatic interactions. Next, excess poly-L-lysine was removed by performing two rinses with Milli-Q H_2_O. Then, 10 µL of small EVs were added onto the treated silica slide. The vesicles were mixed directly on the silica slide with an equal volume of 5% glutaraldehyde solution prepared in PBS to enable vesicle fixation. The mixture was incubated for 30 min. The sample was rinsed and dehydrated with increasing ethanol solutions until it dried to room temperature. A sputter-coating with a thin layer of Au/Pd (about 5 nm thick) of the sample was performed before the measurement in order to ensure the conductivity.

### 3D optical profilometer

The profile and roughness of the samples were measured with a 3D Profilm (Filmetrics) using white light interferometry to analyze the sample surface in 250 µm^2^ × 250 µm^2^ areas with 1 nm in vertical resolution in height. Five different surface regions were measured for each sample and the mean values reported.

### Data processing and statistics

The normality of the data sets was evaluated using Shapiro-Wilk test. Since the data were found to be non-normally distributed, a nonparametric statistical test was applied. Therefore, the significance of data differences was established via the Anova Kruskal-Wallis test (^*^P < 0.05; ^**^P < 0.01; ^***^P < 0.001; ^****^P < 0.0001, respectively). For Cell Vitality assay, since the data sets were found to be normal, statistics were evaluated via Ordinary one-way Anova test (^*^P < 0.05; ^**^P < 0.01; ^***^P < 0.001).

## RESULTS

### PDMS fabrication and characterization

**Figure 1:**
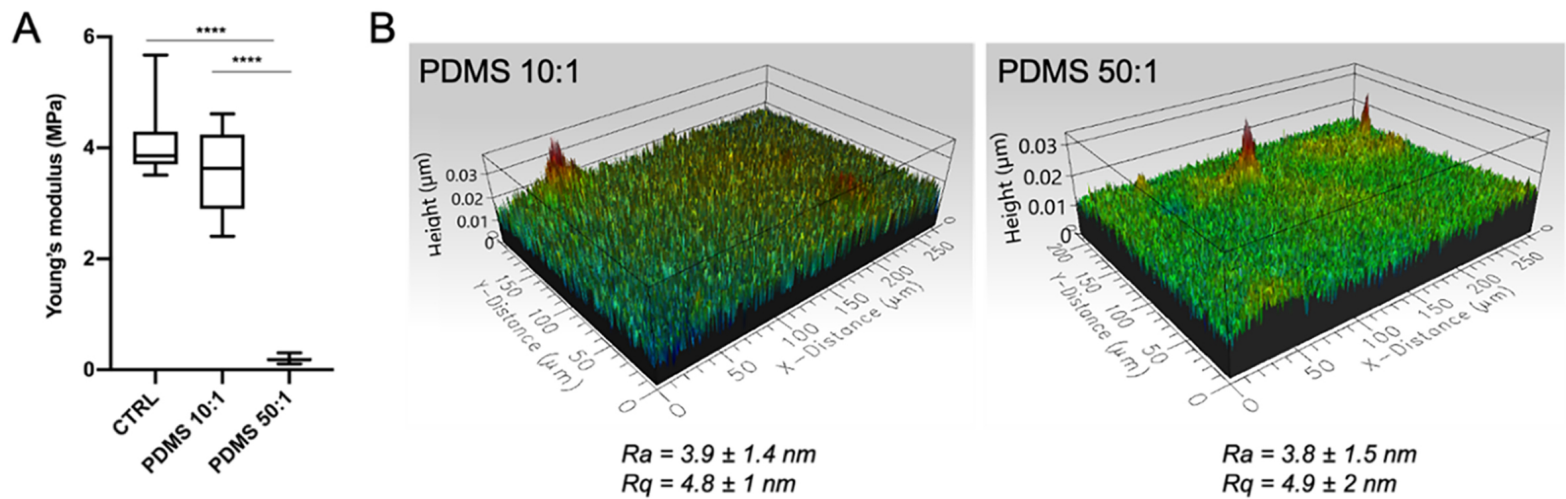
Characterization of PDMS substrates through AFM Force Spectroscopy (A) and 3D optical profilometer system (B). The lower and upper boundaries of the box represent Q1 (25 percentile) and Q3 (75 percentile) of the data, respectively; the horizontal bar inside the box represents the median of the data. Significance of data differences was established via the Anova Kruskal-Wallis test (^*^P < 0.05; ^**^P < 0.01; ^***^P < 0.001; ^****^P < 0.0001, respectively). PDMS: Polydimethylsiloxane; AFM: atomic force microscopy; 3D: three-dimensional.

To investigate the role of substrate stiffness on vesicle release from TNBC cells, we used PDMS, a biocompatible material^[30]^ extensively used to mimic different tissue-like stiffness^[30,31,37]^. PDMS substrates were prepared by mixing the silicone elastomer with the curing agent in different proportions to tune the overall stiffness: 10:1 and 50:1 for stiff and soft PDMS, respectively. Substrate Young’s modulus was characterized through AFM Force Spectroscopy, while both the average roughness (Ra) and root mean square (Rq) were evaluated via a 3D optical profilometer system. Young’s modulus [boxplot in Figure 1A] with average values of 3.6 ± 0.7 MPa and 0.20 ± 0.045 MPa were obtained for stiff (10:1) (from now on referred to as PDMS 10:1) and soft (50:1) (PDMS 50:1) PDMS substrates, respectively. In the case of plastic dish (referred to as CTRL), we measured a stiffness of 4 ± 0.4 MPa. Similar surface roughness was observed between the two different PDMS substrates [3D profilometer images and roughness values in Figure B]; therefore, any differences in cellular behavior will depend mainly on the PDMS stiffness and not on their surface roughness.

### TNBC cells are softer and rounder on soft substrate

**Figure 2:**
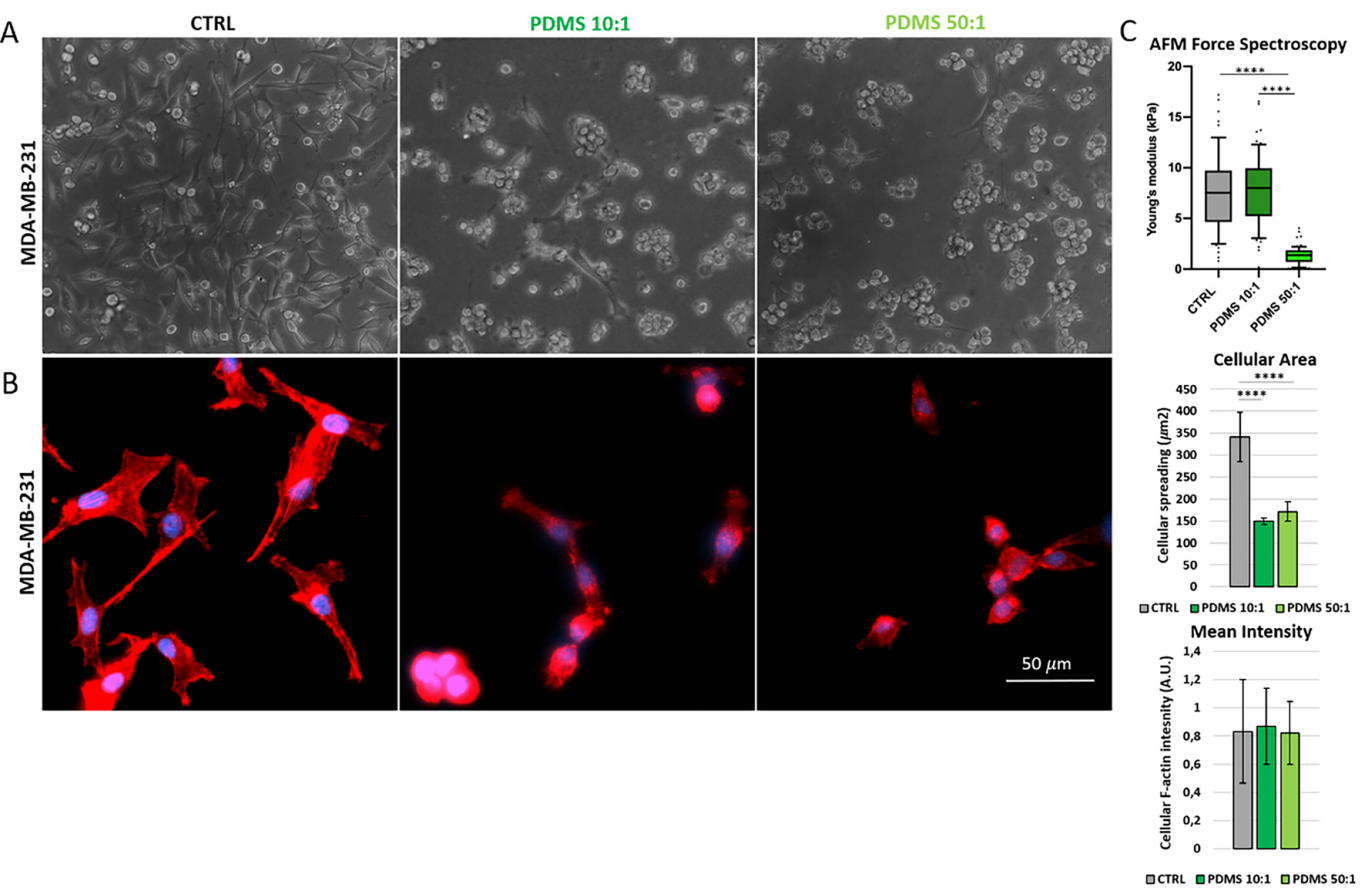
Cell morphology and stiffness of MDA-MB-231 cells plated on different substrates. Representative optical images (A), epifluorescence images with their relative histograms (*n* = 1,123 cells CTRL, *n* = 1,663 cells PDMS 10:1, *n* = 1,853 cells PDMS 50:1) (B), and boxplots showing AFM force spectroscopy results (*n* = 60 cells CTRL, *n* = 63 cells PDMS 10:1, *n* = 62 cells PDMS 50:1) (C) of MDA-MB-231 cells grown on PDMS/without PDMS substrates coated with type I collagen. The lower and upper boundaries of the box represent Q1 (25 percentile) and Q3 (75 percentile) of the data, respectively; the horizontal bar inside the box represents the median of the data. Significance of data differences was established via the Anova Kruskal-Wallis test (^*^P < 0.05; ^**^P < 0.01; ^***^P < 0.001; ^****^P < 0.0001, respectively). PDMS: Polydimethylsiloxane; AFM: atomic force microscopy.

The MDA-MB-231 (TNBC) cells were grown on CTRL, PDMS 10:1, PDMS 50:1 substrates to investigate the respective cell adhesion behavior. To improve the biocompatibility and stabilize cell adhesion on PDMS, the surfaces were coated with type I collagen. Confirming data reported in literature^[38]^, cells are well-spread and elongated on the stiff CTRL substrate and they become semirounded and less spread as the stiffness decreases [shown in optical images of Figure 2A]. The rounded morphology and the relative decrease in the cellular area associated with the substrate softening were also confirmed from the epifluorescence analyses, in which we labeled the cellular F-actin [shown in Figure 2B]. Although there are no significant differences in fluorescence intensity attributable to F-actin, numerous stress fibers in the cells plated on hard dish substrate (CTRL) can be clearly seen from the zoomed epifluorescence images [shown in Figure 2B], compared to the other two conditions. Boxplot graphs showing the values of Young’s modulus of MDA-MB-231 cells in the three different conditions [shown in Figure 2C] faithfully reflect the stiffness trend of the respective substrates, as observed in the literature^[39-41]^.

**Figure 3:**
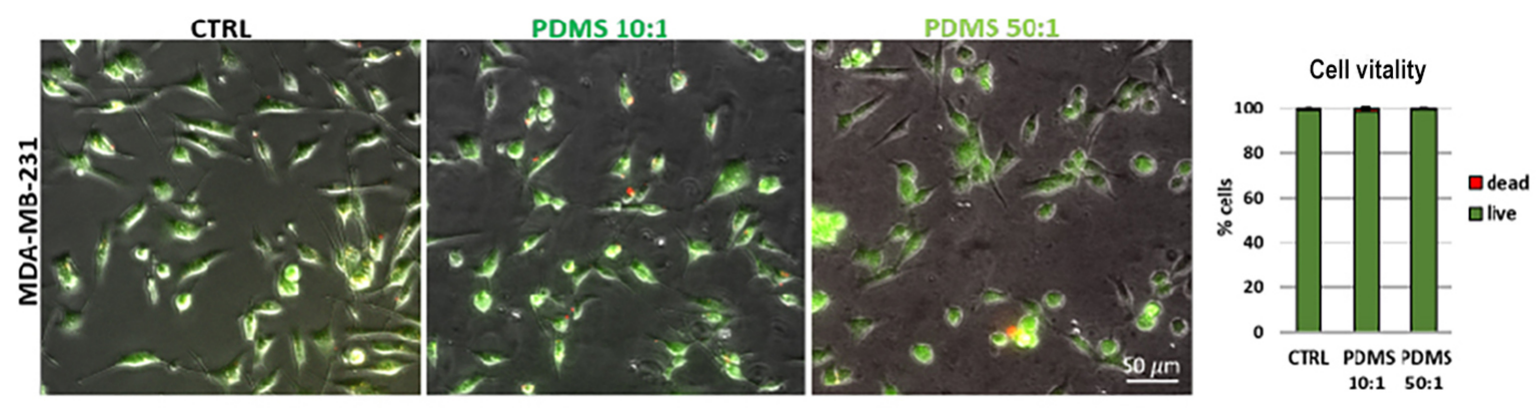
Cell vitality of MDA-MB-231 cells grown on different substrates. Representative epifluorescence images and the relative histogram (*n* = 1,071 cells CTRL, *n* = 932 cells PDMS 10:1, *n* = 1,067 cells PDMS 50:1) showing the live/dead cells of MDA-MB-231 cells plated on dish with or without PDMS. The live cells are identified based on intracellular esterase activity (green), and the dead cells by the lack of esterase activity and non-intact plasma membrane that allows for red dye staining (red). Significance of data differences was established via Ordinary one-way Anova test (^*^P < 0.05; ^**^P < 0.01; ^***^P < 0.001; ^****^P < 0.0001, respectively). PDMS: Polydimethylsiloxane.

The cellular vitality of MDA-MB-231 cells grown on collagen-coated substrates was determined using a live/dead cellular assay. Epifluorescence images [Figure 3] showing the high percentage of live cells (green-labeled) in all three different conditions ensured that any differences in sEV release did not depend on cellular stress or apoptosis induced by the growth on PDMS substrates. No significant differences were observed in the proliferation of MDA-MB-231 cells (evaluated by the counting chamber method) plated on the different substrates [Supplementary Figure 2].

### Soft substrates induce the small extracellular vesicle release in TNBC cells

The isolation and characterization of MDA-MB-231-derived sEVs (referred to as 231_sEVs) were performed as explained in Materials and Methods and as previously reported^[42]^. The isolated vesicles fall within the typical size range of sEVs (from 30 to 200 nm) [Supplementary Figure 3]. As the next step, the characterization of sEVs derived from MDA-MB-231 cells grown on different stiffness substrates was carried out. Aware of the limitations in the extracellular vesicle characterization, mainly due to their small size and heterogeneity^[2]^, orthogonal techniques were used to investigate the physical properties of the 231_sEVs: AFM, NTA, AF4-MALS^[43-45]^.

AFM enables the derivation of label-free, 3D, and semi-quantitative information about isolated sEVs at sub-nanometer scale resolution and under physiological conditions (liquid)^[46]^. Nevertheless, AFM technique for the analysis of sEVs can cause a shrinkage and an artificial cup-shaped morphology of sEVs due to electrostatic interactions (positive charges of Poly-Lysine) used to immobilize them on the surface, and capturing narrow sEV sections of the sample might result in an inaccurate estimation of the EV concentration in solution. Conversely, NTA is a light scattering technique commonly used to determine the concentration and size distribution of sEVs in solution on a single-particle level^[47]^. Unfortunately, this technique also has some limitations. One of them is that, as with other methods based on the Brownian motion principle, larger particles can mask smaller particles, making them undetectable^[48-50]^. Moreover, NTA is based on the light scattering intensity and, for specific composition properties of EVs, it cannot detect vesicles smaller than 60-70 m^[47]^, which instead are identified through the AFM technique. AF4 allows fractionating sEVs by their hydrodynamic size at high resolution in the absence of a stationary phase. The advantages of this technique are the broad size range in which particles can be separated (2-500 nm), reduced risk of shear-induced changes to the sample, good recoveries (> 85%), and high reproducibility^[51]^. When coupled to a multi-angle light scattering detector MALS, information on particle size, size distribution, degradation, and aggregation of the studied particle populations can be obtained^[52]^. AF4-MALS facilitates the successful separation, identification, and collection of distinct subpopulations of sEVs^[53]^. However, this technique fails to detect individual sEVs and to directly measure the sEV concentration.

**Figure 4:**
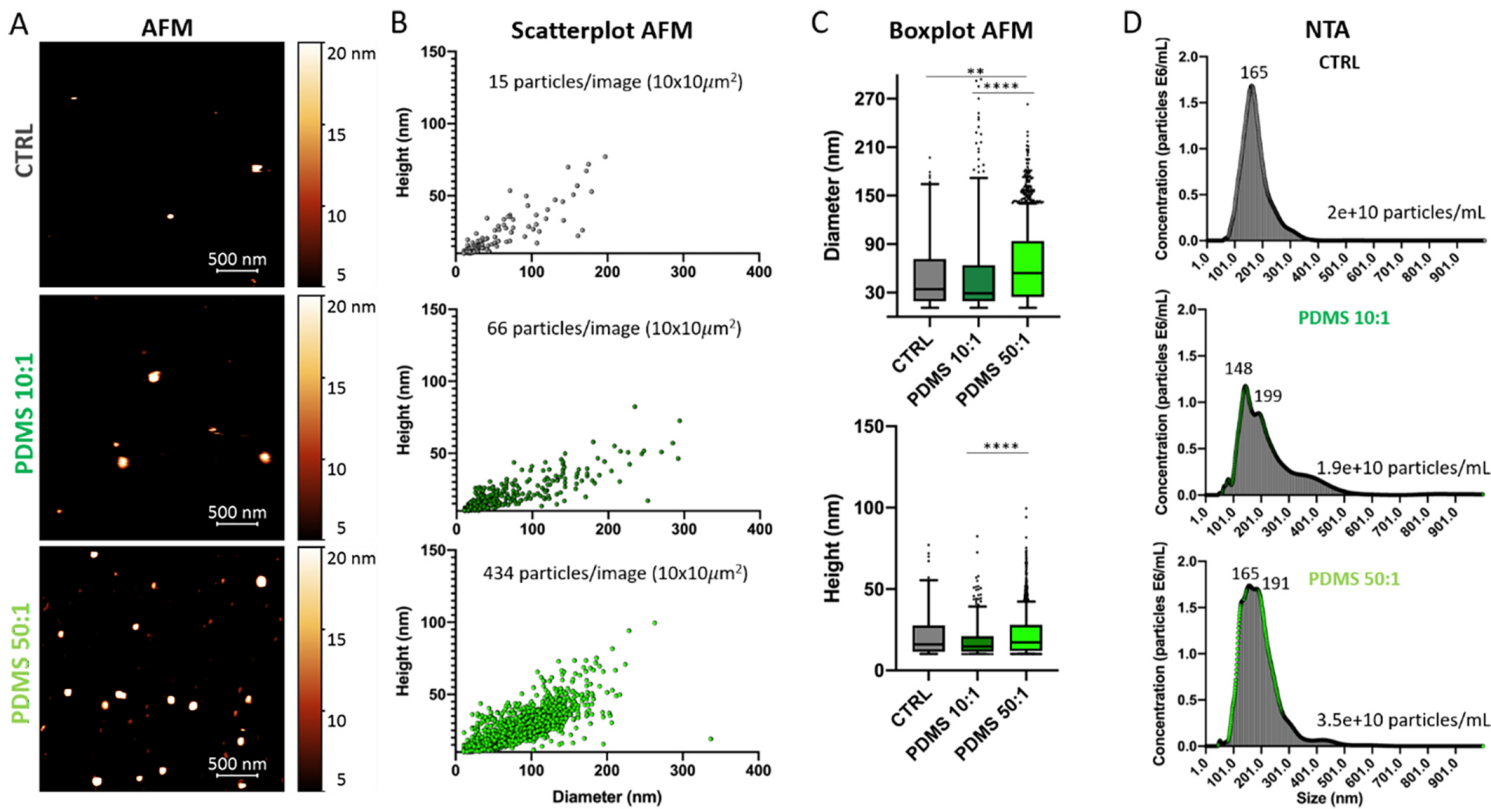
Characterization of 231_sEVs released from cells grown on different substrates. Representative AFM images (A), scatterplots and boxplots obtained from the AFM image analysis (B-C), and NTA graphs of fresh 231_sEVs derived from cells plated on different stiffness substrates. The lower and upper boundaries of the box represent Q1 (25 percentile) and Q3 (75 percentile) of the data, respectively; the horizontal bar inside the box represents the median of the data; NTA results confirmed that 231_sEVs released from cells grown on soft PDMS are more abundant and bigger in size compared to the other conditions. Significance of data differences was established via the Anova Kruskal-Wallis test (^*^P < 0.05; ^**^P < 0.01; ^***^P < 0.001; ^****^P < 0.0001, respectively). Representative experiments of three independent data sets. AFM: Atomic force microscopy; NTA: nanoparticle tracking analysis; PDMS: polydimethylsiloxane; sEVs: small extracellular vesicles.

From now on, CTRL refers to 231_sEVs derived from MDA-MB-231 cells plated on plastic dish, PDMS 10:1 from cells grown on PDMS stiff, and PDMS 50:1 from cells on PDMS soft. AFM imaging in liquid and NTA analyses were performed on fresh (storage at 4 °C for a maximum of one week) 231_sEV samples derived from MDA-MB-231 cells plated on the different stiffness substrates [Figure 4]. AFM images show how 231_sEVs derived from cells plated on PDMS 50:1 substrate appear more abundant and bigger in size than the CTRL and PDMS 10:1 [Figure 4A]. The scatterplots and boxplots reporting the vesicle heights and diameters [Figure 4B-C] obtained from the AFM image analysis confirmed the increase in the number and dimensions of PDMS 50:1 sample compared to other ones. NTA results [Figure 4D] confirmed that 231_sEVs released from cells grown on soft PDMS are more abundant and bigger in size compared to the other conditions. These results highlight that the number and size of vesicles increase by decreasing the substrate stiffness.

**Figure 5:**
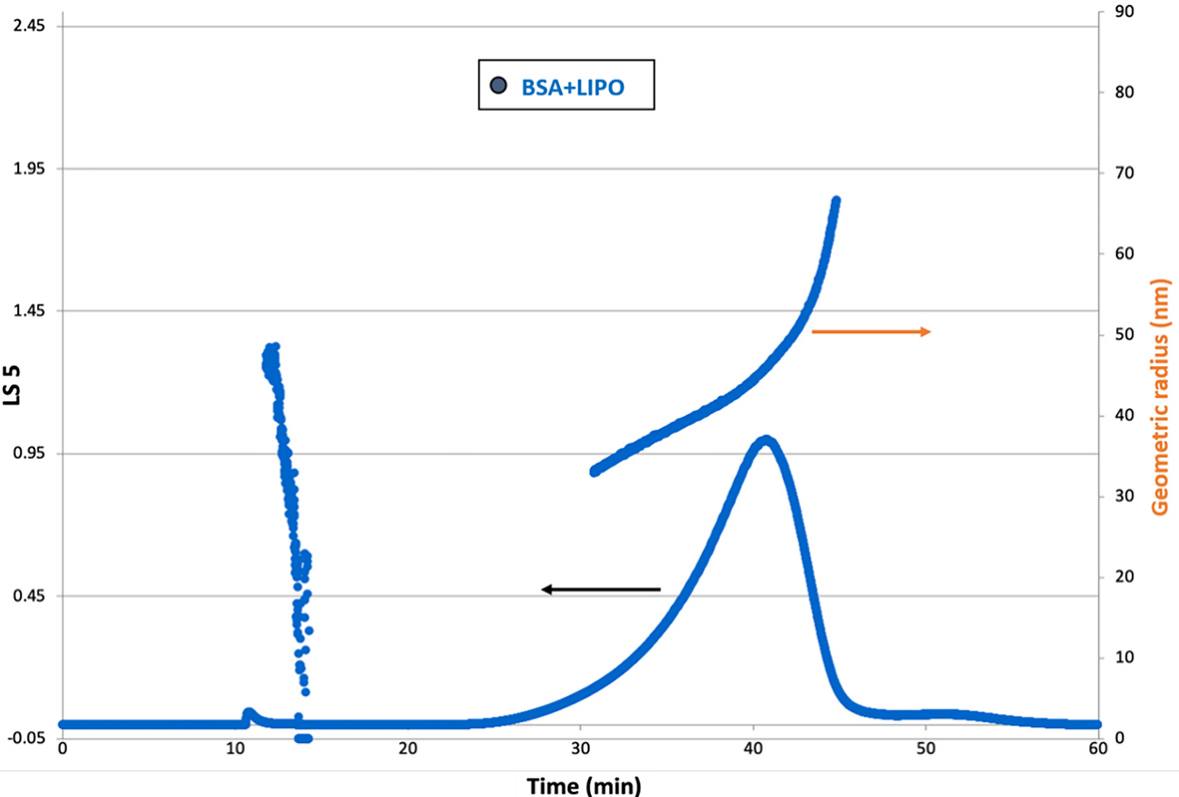
Fractionation and sizing of the reference material (BSA + liposomes) with AF4 coupled to MALS [black arrow: light scattering signal at 90°, orange arrow: geometric radius (nm)]. AF4: Asymmetric flow field-flow fractionation; MALS: multi-angle light scattering; BSA: bovine serum albumin; LIPO: liposomes.

The AF4 separation method was optimized for the analysis of sEVs [shown in Supplementary Figure 4]. The AF4 system was coupled to an 8-angle MALS detector operating with a 658 nm laser and a multi-wavelength detector set to 280 nm. This allowed real-time monitoring and analysis of the vesicles. A solution composed of BSA and liposomes with a known approximate hydrodynamic radius of 40 nm (Lipocure Ltd., Israel, drug-free doxil-like liposomes^[54]^ was analyzed as reference material to check the correct functioning of the method) [Figure 5]. The liposome formulation is made of three different lipids: Hydrogenated Soybean Phosphatidylcholine (HSPC), Methoxy poly(ethylene glycol)-1,2-distearoyl-sn-glycero-3-phosphoethanolamide (MPEG-DSPE), and Cholesterol, mixed at a weight ratio of 57.9:21.9:20.7, as described and well characterized in^[44]^. The fractogram in Figure 5 shows the MALS signal (90° detector angle) on the left axis (continuous line in the graph) and the corresponding geometric radius obtained by applying the ‘coated sphere’ fitting model on the right axis (visualized as big dots in the graph). Liposomes were eluted between 25 and 45 min and their size ranged from 35 to 65 nm in radius, which perfectly matches the real size range of the liposomes. Due to its size, BSA was eluted immediately after the void peak and could not be well-visible from the light-scattering signal but only from the UV absorbance signal [line green in Supplementary Figure 4].

**Figure 6:**
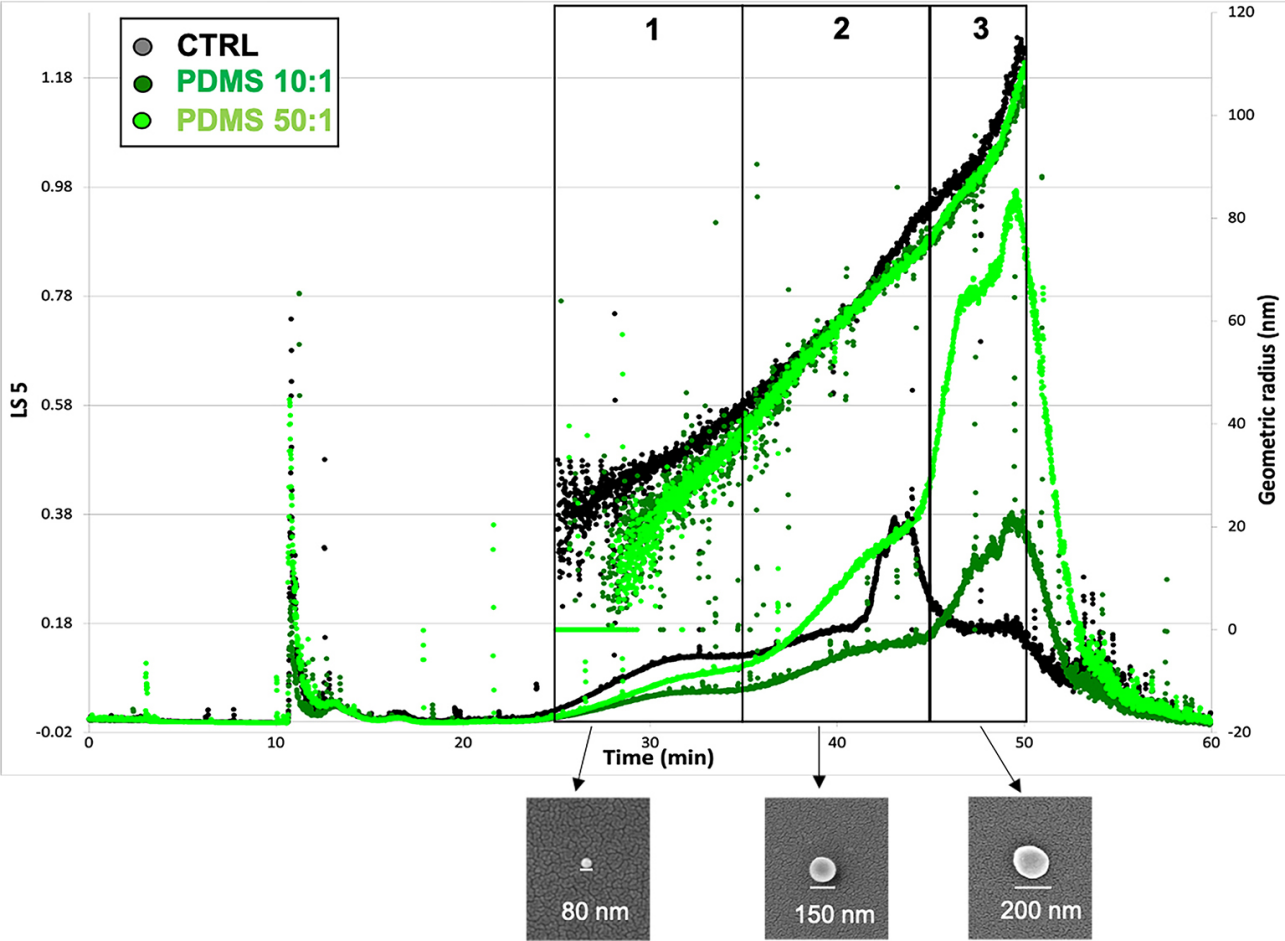
AF4-MALS fractograms showing the three different vesicle subpopulations (1, 2, 3) (on top) and SEM images of the corresponding AF4 fractions (on bottom) of the 231_sEVs derived from MDA-MB-231 cells grown on different substrates. Representative experiment of the three independent data sets. AF4: Asymmetric flow field-flow fractionation; SEM: sanning electron microscopy; PDMS: polydimethylsiloxane; sEVs: small extracellular vesicles.

Several datasets of 231_sEVs both freshly isolated (stored at +4 °C for one week maximum) and stored at -80 °C were measured through AF4-MALS. In the supplementary material [Supplementary Figure 5], we report the AF4 analysis after -80 °C storage, while in Figure 6, the one after 4 °C storage. The ranges in diameter in the two storage cases are the same (as the geometric radius curves show), even though the absolute number of particles is less and the different subpopulations of 231_sEVs are less visible in the -80 °C storage samples compared to the fresh ones; our results confirm the decrease in vesicle number but no significant changes in the diameter after storage at -80 °C, as observed by Robert *et al*.^[44]^. From the comparison of the different 231_sEV samples (CTRL, PDMS 10:1 and PDMS 50:1) after storage at +4 °C [Figure 6], AF4-MALS measurements enabled the identification and differentiation of three different subpopulations of the 231_sEVs, similar to those identified by Zhang *et al*.^[53]^: fraction 1 in the interval from 25 to 35 min distinguishes vesicles from 40 to 80 nm of diameter, fraction 2 from 35 to 45 min vesicles from 80 to 160 nm of diameter, and fraction 3 from 45 to 50 min vesicles from 160 to 200 nm in diameter [shown in Figure 6]. The three vesicle fractions were collected, concentrated again and vesicle sizes of each fraction were confirmed from SEM analyses [Figure 6 bottom]. The major differences were observed in the abundance of each subpopulation: a higher abundance of the smallest subpopulation (fraction 1) was observed in CTRL sample compared to both the PDMS samples; instead, the largest subpopulations (fraction 2 and 3) were more abundant in PDMS 50:1 sample relative to the other conditions. Therefore, these findings corroborated the AFM and NTA results: larger vesicles are released as the substrate stiffness decreases.

## DISCUSSION

TME, which is determined by noncellular and cellular elements, including cancer cells, cancer-associated fibroblasts (CAFs), immune cells, elements of the ECM, and signaling molecules, can significantly influence tumor progression and metastasis^[55]^. The ECM provides the structural framework of the whole TME tissue, consisting of proteins like collagen, elastin, and glycoproteins. Matrix components that can be cross-linked in different ways typically lead to the accumulation of a dense network of matrix molecules accompanied by progressive matrix stiffening in many tumor tissues^[56,57]^. Adhesive and cytoskeletal structures sense both the physical and chemical properties of ECM components, affecting cell adhesion, migration, proliferation, and survival. Cells, in fact, can sense through adhesion complexes and actin-myosin cytoskeleton the physical properties of the surrounding ECM and translate them into biochemical signals to control critical cellular functions that modify their mechanical properties to fulfill novel abilities including migration and proliferation^[58-60]^. Therefore, a change in the stiffness of the TME is a key factor that alters the cell mechanosensing and transduction, directly impacting tumor progression, tumor angiogenesis and metastasis^[56]^. In addition, matrix stiffening also alters cell-cell interactions; in fact, these interactions may be responsible for shifting the phenotypic balance of macrophages residing in TME toward an M2 phenotype favorable to breast tumor progression^[61]^. In terms of stiffness values, in particular, the mammary tissue in healthy conditions is known to be quite soft, measuring a few tens of kPa^[62]^, as measured with AFM. In the case of breast tumor progression, the tissue becomes more heterogeneous, due to the increased production and deposition of ECM components in it. The overall stiffness changes to one hundred KPa^[62]^, close to the soft conditions (PDMS 50:1) exploited in the present work. The variability in biomechanical patterns emphasizes the importance of comprehending the heterogeneity of the extracellular microenvironment and how it governs disease progression in breast cancer.

Among all the elements of TME, EVs also play an important role in cell and TME stiffness. We recently demonstrated that TNBC-derived sEVs can directly modulate the cellular biomechanics of target cells. In detail, sEVs derived from the metastatic MDA-MB-231 cells are able to induce biomechanical rearrangements in non-metastatic MCF7 target cells (stiffer than the previous ones) by decreasing their global cell stiffness^[42]^. Furthermore, we discovered that among all the subcellular elements, chromatin decondensation seems to be the main cause of such EV-induced biomechanical changes^[63]^. In addition, EVs contribute to the tumor cell-mediated activation of CAFs to drive matrix remodeling. Activated CAFs are the principal producers of matrix within solid tumors^[64]^. EVs can directly reprogram CAFs and stromal fibroblasts in primary and metastatic sites by transporting potent fibrogenic signaling activators, including transforming growth factor-β (TGFβ)^[56,65]^. Thus, in our view, it appeared necessary to further explore this crosstalk between TME stiffness and EV release, since both influence each other. We believe that this internal communication within the TME could be the key to cancer cell growth, survival and subsequent metastasis and/or relapse after treatment.

Several works demonstrated that biomechanical properties of healthy and cancer cells can be regulated by modulating the stiffness of the substrates on which cells were cultured (i.e., gelatin, functionalized polyacrylamide or PDMS substrates)^[39,66,67]^. Cells grown on stiff scaffolds have stable focal adhesions, stress fibers, and low motility, while on soft substrates, they show a significant reduction in the cellular elastic modulus^[39-41]^, diffuse and dynamic adhesion complexes, and increased motility^[38,68]^. It is then interesting to see how the release of EVs by these cells might be affected. In terms of the amount of EVs released, only few articles have addressed the issue to date. In particular, Segwick *et al*. observed a higher release of medium EVs from invasive tumor cell lines plated on softer substrates of gelatin compared with the quantity released from the same cells plated on stiffer substrates^[69]^. On the other hand, Wu *et al*. observed an increase in the number of small EVs (but no changes in diameter) on different cancer cell lines, including the breast cancer (MCF-7), MCF10AT (pre-malignant) and MCF10CA (tumorigenic) cells, plated on substrates at different stiffness^[32]^. Wu *et al*. did not include MDA-MB-231 cells in their studies^[32]^, and it has already been observed in other respects that TNBC cells, given their high aggressiveness, metastasis, heterogeneity and relapse after therapy, behave differently compared to other cell lines. For example, some studies have observed that metastatic breast cancer cells, despite the similarities in actomyosin cortex contractility, elasticity, and cell area with the non-invasive/healthy cells, are able to apply normal forces also on soft substrates^[38,70,71]^. Therefore, TNBC might behave and have a different response to the change in matrix stiffness regarding sEV release compared to the other cancer cell lines used by Wu *et al.*^[32]^. Here, we took advantage of a multi-technical characterization approach (AFM, NTA, and AF4-MALS) to carry out a reliable quantitative analysis of the sEVs derived from the metastatic TNBC cells grown on substrates with different stiffness. In particular, AF4-MALS measurements enable the recognition and distinction of different subpopulations of the 231_sEVs from the dimensional point of view. Our results showed that MDA-MB-231 cells, when plated on both the PDMS scaffolds, despite the similarity in actin properties, are rounder than on plastic dish, and their stiffness is significantly decreasing only on the soft PDMS. Moreover, our findings, which corroborate the study of Segwick *et al*.^[69]^, revealed that MDA-MB-231 cells plated on softer scaffolds release a higher number and larger subpopulations of sEVs compared to those on stiffer substrates. It is well known from the literature that the physical properties of the substrate influence the adhesions of cells, which can then transmit forces and modulate the cytoskeleton network^[72]^, affecting trafficking to plasma membrane and changes in cellular lipid composition and fluidity^[73]^. We, therefore, make the hypothesis that the increase in sEV release observed in MDA-MB-231 cells plated on lower stiffness substrates is due to these switches in focal adhesions, cytoskeleton, and plasma membrane properties. Indeed, ECM stiffness can affect different pathways (e.g., integrin-FAK-PI3K-Akt, YAP/TAZ, AMPK, and Rho/Rock-actin cytoskeleton), which regulate the synthesis of cellular fatty acids and cholesterol and the translocation of the fatty acid transporter CD36 to the cell membrane^[74]^. We hypothesize that this mechanism could underlie the survival of TNBC cells at various stages of TME inhibition. Indeed, TME has an antitumor function at the early stage of tumor development, but some tumor cells can tolerate this inhibition and reprogram TME into a pro-tumor environment^[56]^. So, as metastatic tumor cells are able to apply normal forces even on soft substrates^[38,70,71]^, this additional mechanism of increased sEV release might represent a mechanism of surviving the various inhibition controls. Furthermore, the increase in sEV release with decreasing cell stiffness, as for metastatic cancer cells, could also represent a further means of spreading of, for instance, TNBC cells, which colonize soft secondary tumoral sites (e.g., brain and liver)^[75]^. Nevertheless, the relationship between ECM stiffness and TNBC progression and metastasis through EVs must be further examined, especially from a molecular point of view.

In conclusion, in this study, we primarily investigated the release of EVs from MDA-MB-231 cells cultured on collagen-coated substrates with varying stiffness. We demonstrated that substrates with stiffnesses similar to that of breast tumor tissues (around 200 kPa) induce changes in Young’s modulus and the morphology of cultured cells, leading to the release of a higher number of vesicles with larger diameters. This finding reveals an indirect regulation of EVs by ECM stiffness, providing valuable insights that could be crucial in combating the metastatic spread of TNBC. We anticipate that other ECM components, such as fibronectin, may also significantly influence cell adhesion, invasiveness, and EV release^[76]^. Future research will aim to extend our multi-technique approach to evaluate the key molecular contributions of different ECM components to overall ECM stiffness and their impact on the compositional changes in EVs. We also emphasize the importance of validating our findings on additional, independent TNBC cell lines.

## DECLARATIONS

### Availability of data and materials

Data supporting the present findings are available upon reasonable request.

### Copyright

© The Author(s) 2024.
