# Supplementary material for: Substrate stiffness modulates extracellular vesicles’ release in a triple-negative breast cancer model
Source: Extracell Vesicles Circ Nucl Acids. 2024 Sep 29;5(3):553–68. doi: 10.20517/evcna.2024.47 (PMC11648499; doi:10.20517/evcna.2024.47)
Supplement: Supplementary file 1 [file evcna-5-3-553-SupplementaryMaterials.pdf]

Supplementary Materials

Substrate stiffness modulates extracellular vesicles' release in a triple-negative breast cancer model

Beatrice Senigagliesi[1,2,3], Otmar Geiss[3], Stefano Valente[3,4], Hendrik Vondracek[2], Nicola Cefarin[5], Giacomo Ceccone[3], Luigi Calzolai[3], Laura Ballerini[1], Pietro Parisse[2,5], Loredana Casalis[2]

[1]Neuroscience Area, Scuola Internazionale Superiore di Studi Avanzati, Trieste 34136, Italy.
[2]Nano-Innovation Laboratory, Elettra-Sincrotrone Trieste S.C.p.A., Trieste 34149, Italy.
[3]Institute for Health and Consumer Protection, European Commission - Joint Research Centre, Ispra 21027, Italy.
[4]Department of Physics, University of Trieste, Trieste 34127, Italy.
[5]Istituto Officina dei Materiali, Consiglio Nazionale delle Ricerche, CNR-IOM, Trieste 34149, Italy.

Correspondence to: Dr. Loredana Casalis, Dr. Pietro Parisse, Nano-Innovation Laboratory, Elettra-Sincrotrone Trieste S.C.p.A., s.s. 14 km 163,5 in Area Science Park, Trieste 34149, Italy. E-mail: Loredana.casalis@elettra.eu; parisse@iom.cnr.it;
Dr. Laura Ballerini, Neuroscience Area, Scuola Internazionale Superiore di Studi Avanzati, Via Bonomea 265, Trieste 34136, Italy. E-mail: ballerin@sissa.it;
Dr. Luigi Calzolai, Institute for Health and Consumer Protection, European Commission - Joint Research Centre, Via Enrico Fermi 2749, Ispra 21027, Italy. E-mail: CALZOLAI@ec.europa.eu

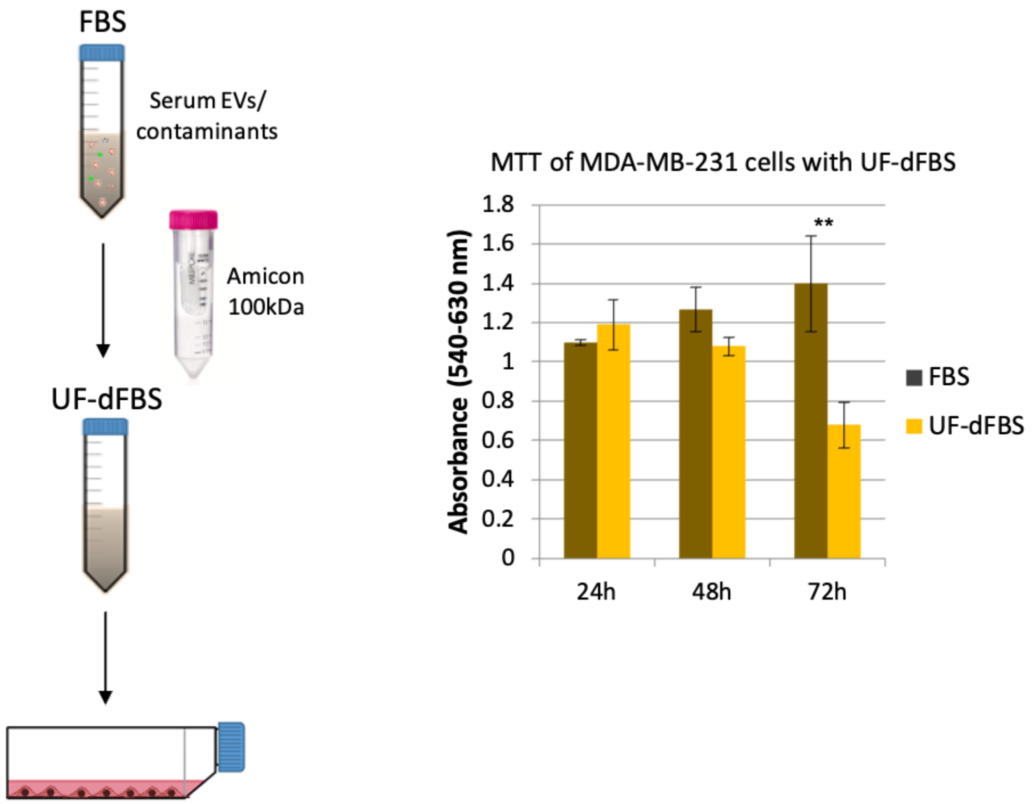

Supplementary Figure 1. MTT assay showing the cell vitality of MDA-MB-231 cells grown with UF-dFBS for 1,2,3 days. Significance of data differences was established via two-tailed Student's t-test. UF-dFBS： Ultrafiltrated EV-depleted FBS.

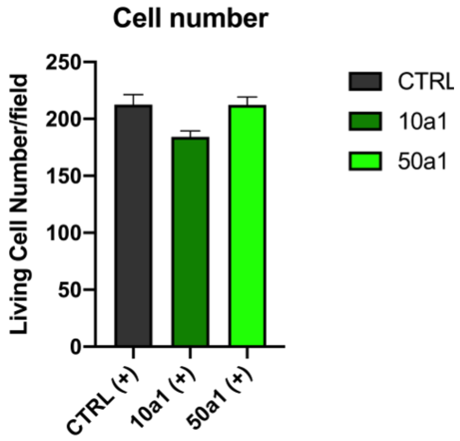

Supplementary Figure 2. Cell Number assay showing the cell proliferation of MDA-MB-231 cells grown on substrates at different stiffness. Significance of data differences was established via Ordinary one-way Anova test.

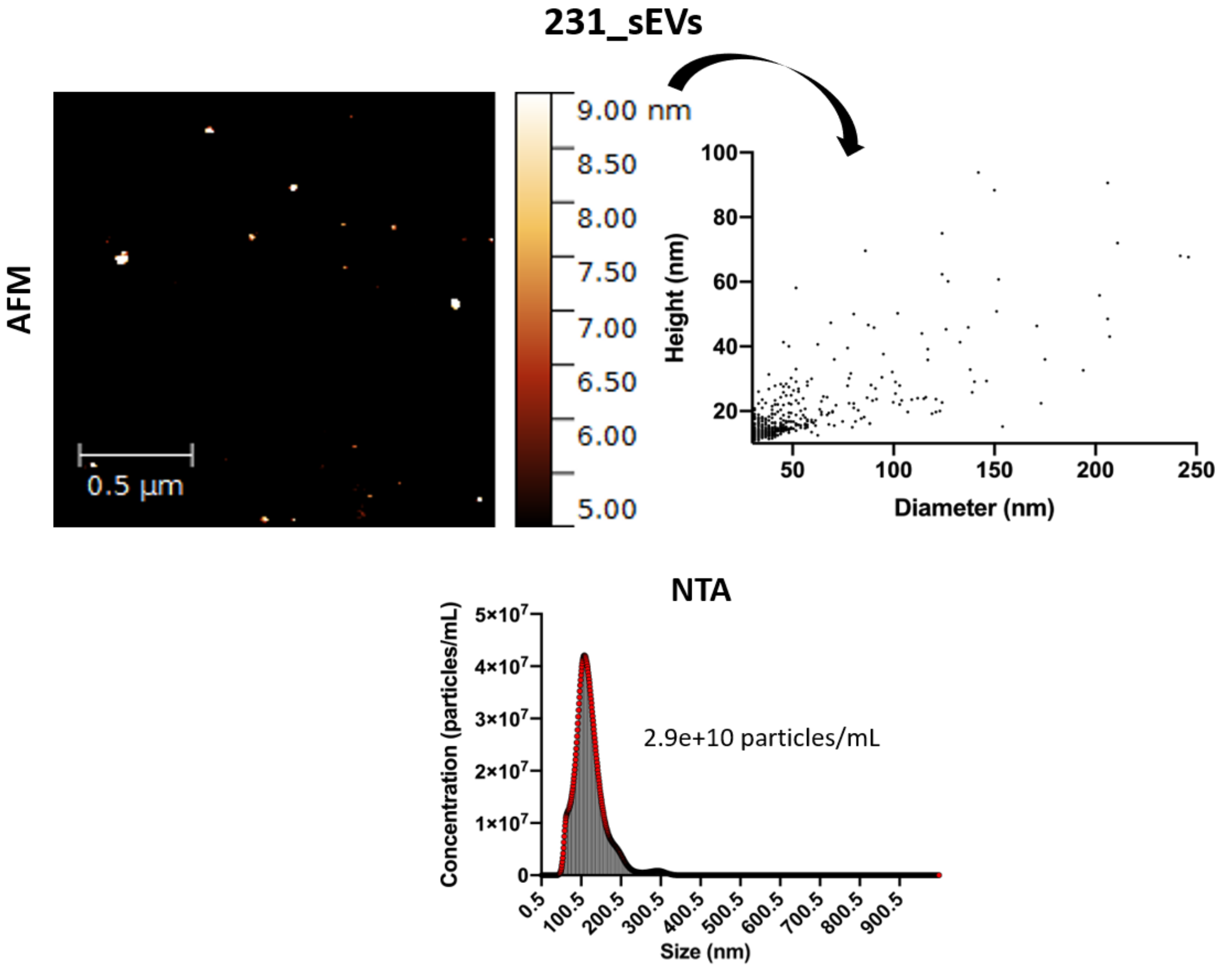

Supplementary Figure 3. MDA-MB-231-derived sEV (231_sEVs) characterization: a representative atomic force microscopy image, scatterplot showing height and diameter obtained from the analysis of AFM images, and NTA. AFM: Atomic force microscopy; NTA: nanoparticle tracking analysis.

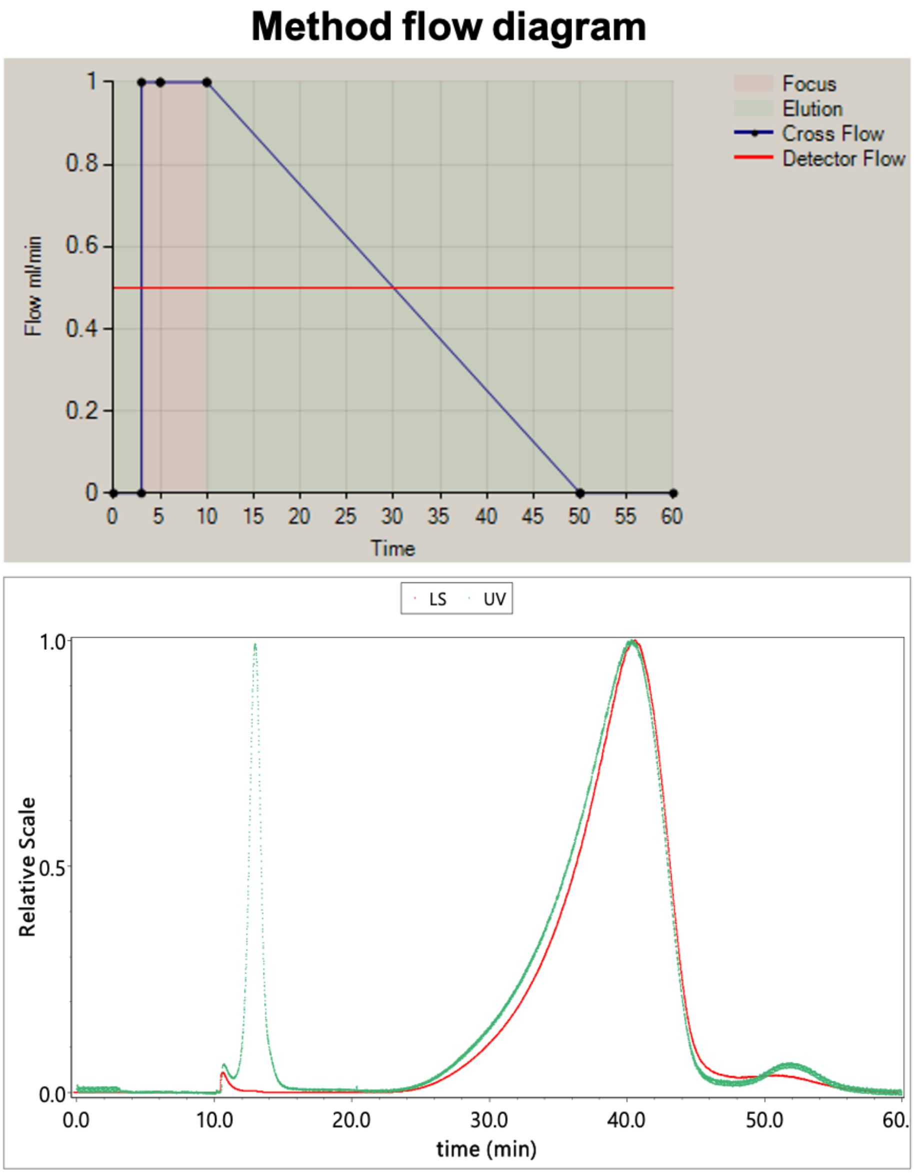

Supplementary Figure 4. Method flow diagram used for the AF4-MALS measurements and the AF4-MALS fractogram of the reference material (BSA + liposomes) using this method (on bottom). The graph shows the MALS signal (90° scattering angle, LS 5) in red and the UV absorbance at 280 nm in green. AF4: Asymmetric flow field-flow fractionation; MALS: multi-angle light scattering.

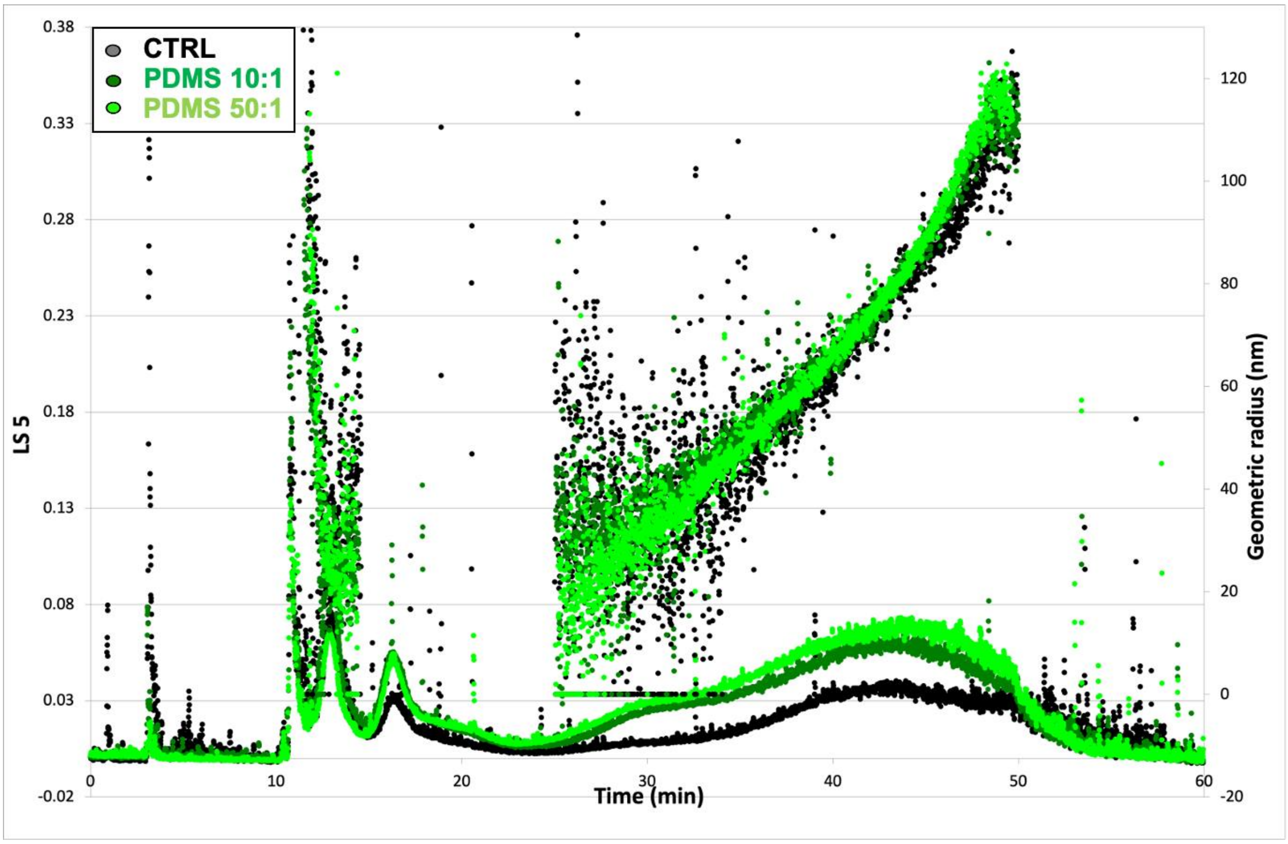

Supplementary Figure 5. AF4-MALS fractograms of 231_sEVs stored at -80°C. AF4: Asymmetric flow field-flow fractionation; MALS: multi-angle light scattering.
